# Expression of *OCT4* isoforms is reduced in primary colorectal cancer

**DOI:** 10.3389/fonc.2023.1166835

**Published:** 2023-06-20

**Authors:** Eva Turyova, Peter Mikolajcik, Marian Grendar, Eva Kudelova, Veronika Holubekova, Michal Kalman, Juraj Marcinek, Matej Hrnciar, Michal Kovac, Juraj Miklusica, Ludovit Laca, Zora Lasabova

**Affiliations:** ^1^ Department of Molecular Biology and Genomics, Jessenius Faculty of Medicine in Martin, Comenius University Bratislava, Martin, Slovakia; ^2^ Clinic of Surgery and Transplant Center, Jessenius Faculty of Medicine in Martin and University Hospital Martin, Comenius University Bratislava, Martin, Slovakia; ^3^ Biomedical Centre Martin, Jessenius Faculty of Medicine in Martin, Comenius University Bratislava, Martin, Slovakia; ^4^ Department of Pathological Anatomy, Jessenius Faculty of Medicine in Martin and University Hospital Martin, Comenius University Bratislava, Martin, Slovakia; ^5^ Department of Informatics, Information Systems and Software Engineering, Faculty of Informatics and Information Technologies, Slovak University of Technology, Bratislava, Slovakia

**Keywords:** colorectal cancer, gene expression, oct4, isoforms, carcinogenesis

## Abstract

**Introduction:**

Colorectal cancer (CRC) is one of the most common types of cancer worldwide. The carcinogenesis of CRC is indeed complex, and there are many different mechanisms and pathways that contribute to the development of malignancy and the progression from primary to metastatic tumors. The OCT4A, encoded by the *POU5F1* gene, is a transcription factor responsible for the phenotype of stem cells, maintaining pluripotency and regulation of differentiation. The *POU5F1* gene is made up of five exons that can create numerous isoforms through alternative promoter or alternative splicing. In addition to *OCT4A*, other isoforms called *OCT4B* are also translated into protein; however, their role in cells has been unclear. The aim of our work was to investigate the expression patterns of *OCT4* isoforms in primary and metastatic CRC, providing us with useful information about their role in the development and progression of CRC.

**Methods:**

Surgical specimens from a total of 78 patients were collected and isolated from primary tumors (*n* = 47) and metastases (*n* = 31). The relative gene expression of *OCT4* isoforms was investigated using the RT-qPCR method together with the TaqMan probes for particular *OCT4* isoforms.

**Results:**

Our results suggest significantly downregulated expression of the *OCT4A* and *OCT4Bs* isoforms in both primary (*p* = 0.0002 and *p* < 0.0001, respectively) and metastatic tumors (*p* = 0.0006 and *p* = 0.00051, respectively) when compared with the control samples. We also observed a correlation between reduced expression of all *OCT4* isoforms and both primary and left-sided tumors (*p* = 0.001 and *p* = 0.030, respectively). On the other hand, the expression of all *OCT4* isoforms was significantly upregulated in metastases compared with primary tumors (*p* < 0.0001).

**Discussion:**

Unlike previous reports, we found out that the expression of *OCT4A*, *OCT4Bs*, and all *OCT4* isoforms was significantly reduced in primary tumors and metastases compared with control samples. On the other hand, we supposed that the expression rate of all *OCT4* isoforms may be related to the cancer type and side, as well as to liver metastases. However, further studies are required to investigate the detailed expression patterns and significance of individual *OCT4* isoforms in carcinogenesis.

## 1 Introduction

Colorectal cancer (CRC) is one of the most common types of cancer worldwide. The global statistics from 2020 show that colorectal cancer creates 10% of all newly diagnosed cases, which means that after breast and lung cancer, it is the third most common type (1). Regardless of the enormous interest in CRC research, the annual incidence and number of CRC-related deaths have been increasing worldwide. The incidence of CRC is higher in developed countries due to unhealthy lifestyle including high consumption of red meat and alcohol, smoking, sedentary lifestyle, and inflammatory bowel diseases (2–4).

The octamer binding transcription factor 4 (*OCT4*) isoforms are encoded by the *POU5F1* gene which is located at the short arm of chromosome 6 (5). Proteins from the POU protein family contain the so-called POU domains which allow them to bind to DNA and influence the gene expression, as well as interact with other transcription factors and cofactors. The POU domain recognizes and binds to the octamer consensus DNA sequence ATGCAAT, and in this way, the OCT4 can regulate gene expression, making it the main regulator of maintaining pluripotency and self-renewal of the stem cells (6–8). The human *OCT4* gene can, through alternative transcription initiation or alternative splicing, create numerous different isoforms. These isoforms differ not only in nucleotide sequence but also in subcellular localization and properties. At the RNA level, *OCT4* creates four groups of variants, namely, *OCT4A*, *OCT4B*, *OCT4C*, and *OCT4D*, and each of them uses a unique transcription start site. Furthermore, the mechanism of alternative splicing is responsible for the emergence of other *OCT4B* and *OCT4C* isoforms (9, 10). The discovery of individual transcripts and isoforms was gradual, and to date, overall, 10 *OCT4* transcripts have been identified (9, 11–16). Not all transcripts have been identified also at the protein level, and there are only assumptions that the length of the protein product would be 164 amino acids (16). On the other hand, there is unequivocal evidence that the *OCT4A* and *OCT4B* variants generate distinct protein products that differ in their properties. The *OCT4A* isoform encodes the longest protein composed of 360 amino acids, which fulfills the role of the transcription factor. In contrast, the *OCT4B* transcript may be through the mechanism of alternative translation translated into three different proteins with lengths of 265, 190, and 164 amino acids (17). *OCT4B* proteins differ from *OCT4A* in DNA-binding properties; as a consequence of the inability of *OCT4B* proteins to regulate gene expression, their role in the cell remains unknown (18).

The *OCT4A* is the most known isoform, and its correct and precise expression is necessary for the regulation of the expression of further genes, as well as for the repression of genes involved in differentiation (18). The OCT4A protein plays a pivotal role in pluripotency and its expression was confirmed in embryonic stem (ES) and embryonic cancer (EC) cells, tumor cells, and cell lines (19, 20), but its presence has not yet been detected in non-pluripotent cell types (11, 18). The definite role of OCT4A in pluripotent cells was clarified by Takahashi and Yamanaka who proved that OCT4 is one of the four transcription factors needed for the reprogramming of somatic cells to pluripotent cells (12, 13, 21). In contrast to OCT4A, which is located exclusively in the nucleus, the OCT4B isoform is diffusely localized in both the cytoplasm and nucleus (17, 22). Atlasi et al. demonstrated that *OCT4B* is expressed in almost all cell types tested, including ES, EC, and somatic cell types (12). Interestingly, another identified *OCT4B* isoform, called *OCT4B1*, has properties and expression patterns more similar to the *OCT4A* than to the *OCT4B* isoform.

The role of *POU5F1*, especially the *OCT4A* isoform in stem cells, emphasizes its potential role in carcinogenesis. The so-called theory of stem cells in carcinogenesis claims that cancer stem cells are capable of inducing tumorigenesis and tumor growth due to their self-renewal ability, as well as giving rise to additional progenitor cells (23). Due to the great diversity of *OCT4* isoforms, little is known about their expression patterns and role in primary and metastatic colorectal cancer. In our study, we focused on determining the expression patterns of *OCT4A*, *OCT4Bs*, and all *OCT4* isoforms in primary and metastatic colorectal cancer, as well as the correlation between their expression and clinical parameters.

## 2 Methods

### 2.1 Clinical tissue samples

In total, 78 CRC patients were registered and underwent CRC resection. Primary tumor samples as well as metastatic samples were collected in collaboration with the University Hospital Martin, the Clinic of General, Visceral and Transplant Surgery, and the Department of Pathological Anatomy (Martin, Slovakia). Control, adjacent non-tumorous samples were obtained from patients with primary tumor (*n* = 30). The inclusion criteria were confirmation of diagnosis by histopathological examination, TNM classification, and clinical stages I, II, and III for primary tumor samples (*n* = 47) and confirmation of diagnosis by histopathological examination, TNM classification, and clinical stage IV for samples of liver metastases (*n* = 31). The pathologist also decided to take a non-cancer control sample from the adjacent tissue as far as possible from the tumor. The exclusion criteria were age less than 40 years or the simultaneous presence of other cancer types or liver metastasis of unknown origin. The histopathological assessment such as staging, grading, and typing of tumors was done by experienced pathologists (MK and JM). The clinicopathological characteristics of the patients included in the study are listed in **Table 1**. Tumor surgical specimens were collected by the pathologists and on the same day embedded into a solution of Dulbecco’s modified Eagle’s medium (DMEM), fetal bovine serum (10%), and penicillin/streptomycin and stored at 4°C. Immediately, the samples were frozen in RNAlater and then transferred to the Department of Molecular Biology and Genomics for RNA isolation.

**Table 1:** Clinicopathological characteristics of the patients included in the study.

	Primary tumor samples	Liver metastases samples
**Patients**	47	31
**Females**	20	8
**Males**	27	23
**Average age**	66.7 (SD ± 11.2)	67.6 (SD ± 9.1)
**BMI**	27.6 (SD ± 5.3)	28.1 (SD ± 4.9)
Grade		
**G1**	**G2**	**G3**	**G n.a.**	13 21 9 4	1 16 3 11
T stage		
**T1**	**T2**	**T3**	**T4**	**T n.a.**	2 10 25 10 0	1 5 20 2 3
N stage		
**N0**	**N1**	**N2**	**N n.a.**	28 13 6 0	7 14 6 4
Clinical stage		
**I**	**II**	**III**	**IV**	9 20 14 4	0 0 0 30

n.a, not available.

### 2.2 RNA purification and cDNA preparation

Total RNA was purified using the RNeasy Mini Kit (Qiagen, Hilden, Germany) according to the manufacturer’s instructions, treated with DNase, and stored at −80°C. Total RNA was quantified using the Qubit 3.0 Fluorometer (Thermo Fisher, MA, USA), and RNA quality assessment was performed on Agilent Bioanalyzer 2100 (Agilent Technologies, Santa Clara, USA). Reverse transcription was performed for each sample using a High-Capacity cDNA Reverse Transcription Kit with RNase inhibitor (Thermo Fisher, MA, USA) according to the manufacturer’s instructions in a total volume of 20 µl consisting of 2 µl of 10× RT buffer, 0.8 µl of 2× dNTP mix (100 mM), 2 µl of 10× RT random primers, 1 µl of MultiScribe™ Reverse Transcriptase, 1 µl of RNase inhibitor, 3.2 µl of nuclease-free water, and 10 µl of RNA diluted in nuclease-free water to an overall concentration of 500 ng/µl. Reverse transcription was performed using the Bio-Rad MJ Mini Personal Thermal Cycler (Bio-Rad, Hercules, CA, USA), and the thermal conditions were as follows: 25°C for 10 min, followed by 37°C for 120 min and 85°C for 5 min. The prepared cDNA was stored at −20°C.

### 2.3 Relative quantification of the gene expression

For the RT-PCR analysis, 10 µl of Gene Expression Master Mix was mixed with 1 µl of *POU5F1* TaqMan assay, 1 µl of *GAPDH* TaqMan assay, and 1 µl of cDNA. The total volume of 20 µl per reaction was supplemented with nuclease-free water. Expression analysis was performed by real-time quantitative PCR (qPCR) using LightCycler ABI 3500 (Applied Biosystems, Foster City, CA, USA), TaqMan Gene Expression Master Mix, and several TaqMan assays (Thermo Fisher, MA, USA) for specific *OCT4* isoforms and endogenous control. We determined the expression of the *OCT4A* isoform, *OCT4B* isoforms, and all *OCT4* isoforms together using three different TaqMan assays (FAM-MGB) (assay ID Hs01654807_s1, Hs04195369_s1, and Hs04260367_gH, respectively). Individual assays were chosen based on genomic map analysis (**Supplementary Figure 1**) according to the manufacturer, as well as based on the relevant literature. The chosen assays have already been validated and used in several articles, for instance, Hs04260367_gH (24, 25), Hs01654807_s1 (26, 27), and Hs04195369_s1 (28, 29). The assay used for the detection of the OCT4A isoform did not recognize other OCT4 isoforms due to its exon 1 specificity. The same principle was used in the experiment with assay specific for almost all OCT4B isoforms. Some of the OCT4B and OCT4A isoforms were not recognized by this assay due to the absence of a certain exon. The last assay was used to determine all OCT4 isoforms that share the same last exon (**Supplementary Figure 1**). Glyceraldehyde-3-phosphate dehydrogenase (GAPDH) was used as an endogenous control, and its expression was determined by TaqMan assay (VIC-MGB, assay ID Hs99999905_m1), too. As a reference sample, we used the Total Human RNA Control (Thermo Fisher, MA, USA; Cat. number 4307281) which was also reverse-transcribed into cDNA. The thermal conditions of the reactions were as follows: 50°C for 2 min, 95°C for 10 min, followed by 40 cycles at 95°C for 15 s and 60°C for 60 s. All reactions were performed in duplicate. Contamination was controlled by using a no-template control without adding cDNA as a template molecule. The relative quantification of *OCT4* isoform expression in the primary and metastatic tumor samples but also in adjacent non-tumorous tissue was performed by the ΔΔCt method (30). We calculated the relative expression of *OCT4A*, *OCT4B*, and all *OCT4* isoforms against the GAPDH expression separately and obtained a fold-change value (log2FC). For statistical analysis, log2(FC) values were used.

### 2.4 Statistical analysis

Data were explored and analyzed in collaboration with the Biomedical Centre Martin in R ver. 4.0.5, with the aid of different libraries (31–41). Data were summarized as the mean, SD, min, quartiles, and max. Boxplot overlaid with a swarm plot and quantile–quantile plot with the 95% confidence band constructed by bootstrap were used to assess the normality of data. Welch’s *t*-test was used to test the null hypothesis that the population mean of the log2(FC) is 0. The regression model was used to model the association between log2(FC) and clinical data. Using the Wilkinson–Rogers notation, the full model that we used can be written as log2FC ~ type + side + T + N + M + stage, where type is a factor with levels *p* (for primary tumor) and *t* (for metastasis); side is a 0/1 factor where the left side is coded as 1; T, N, and M are the factors, describing the amount and spread of cancer; and stage is the pathological stage. The Akaike information criterion (AIC) was used for model selection. The model selected by the AIC was subjected to standard diagnostic analyses. Adjusted *R* ^2^ was used to measure the effect size. Marginal means and *post hoc* pairwise comparisons were performed where the AIC-selected model contained other predictors aside from the intercept. The *post hoc p*-values were adjusted by the Tukey method.

### 2.5 TCGA data

The STAR-processed RNA sequencing level 1 data were downloaded from the GDC Portal of the NIH National Cancer Institute. Files were generated within the projects TCGA-COAD, TCGA-READ, CPTAC-2, and HCMI-CMDC. The set contained tsv files of 737 samples from 616 individuals, of which 615 were tumor samples, 45 were labeled as normal tissue, and 11 were labeled as metastases. Sixty-six samples with no information of their origin were omitted from further analysis. Given that all files were open access level 1 data, no preprocessing was carried out.

## 3 Results

In our study, we focused on determining the expression of different *OCT4* isoforms in primary and metastatic colorectal cancer samples. We investigated the expression of the *OCT4A* isoform, *OCT4B* isoforms, and all *OCT4* isoforms together, as well as the relationship between isoform expression in certain sample types and clinical data. A total of 78 cancer samples (47 primary cancer samples and 31 liver metastases samples) were analyzed in duplicates for each isoform (A, B, and all isoforms). Control samples (*n* = 30) represented adjacent non-tumor tissues of the intestinal epithelium.

### 3.1 Relative quantification of the gene expression

**Figure 1:**
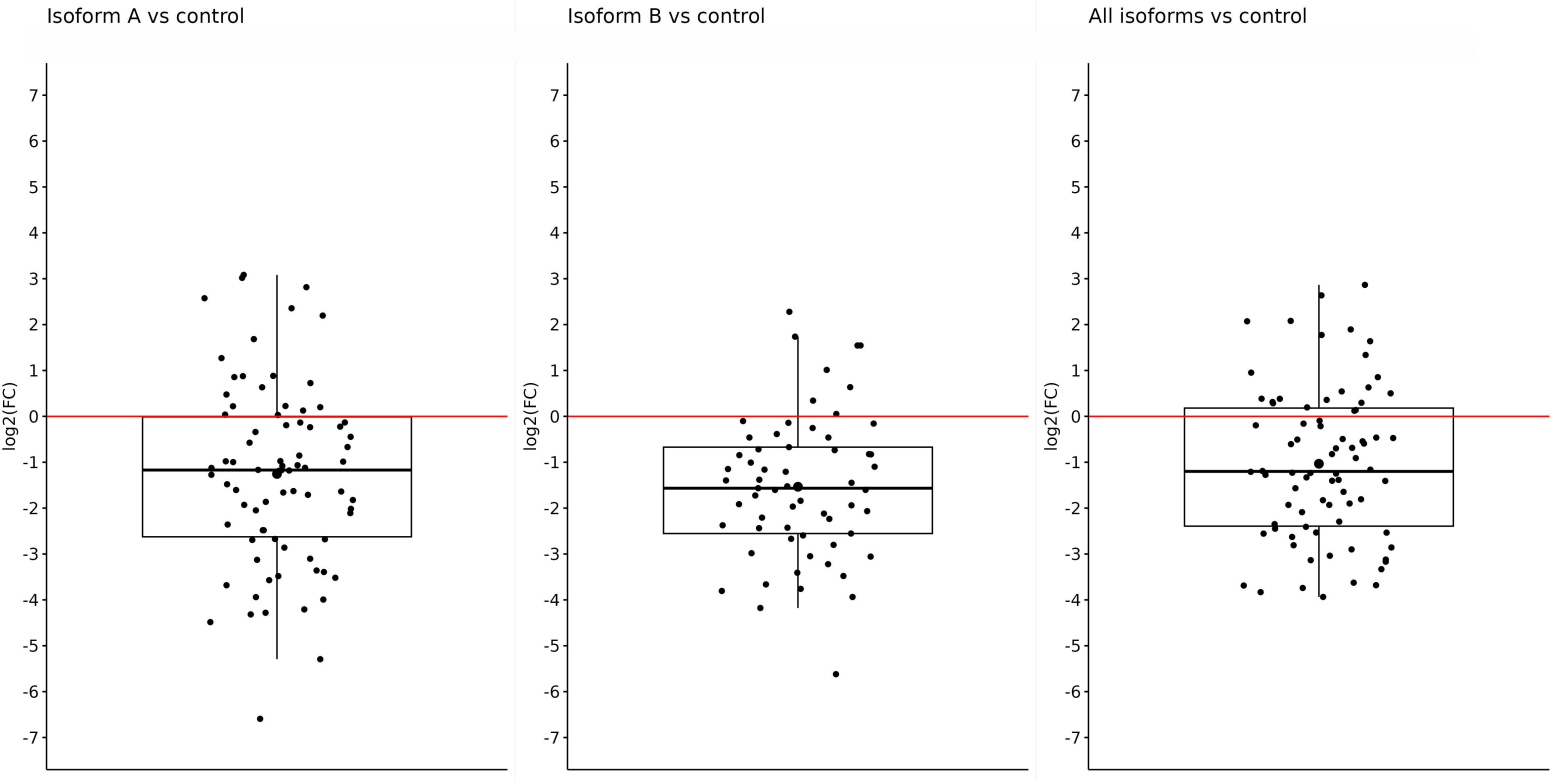
Expression of *OCT4A*, *OCT4B*, and all *OCT4* isoforms in tumor samples (primary + metastatic) compared with control samples. On the *y*-axis, we see log2(FC) values for OCT4A (−1.25), OCT4B isoforms (−1.53), and all OCT4 isoforms (−1.03), which means a significantly reduced expression of all mentioned isoforms in primary tumors compared with control.

Fold-change values were calculated based on qPCR results for individual isoforms in specific sample types. At first, we compared the expression of the *OCT4* isoform in all obtained tumor samples (primary + metastatic) *vs*. control (adjacent non-tumor tissue). The expression of all tested isoforms (namely, *OCT4A*, *OCT4B*, and all *OCT4* isoforms) was significantly downregulated in tumor tissues compared with controls. The log2(FC) values for the *OCT4A* and *OCT4B* isoforms were −1.25 and −1.53, respectively, which refers to significantly reduced expression in tumor samples compared with controls (*p* < 0.0001 and *p* < 0.0001, respectively). In the case of all *OCT4* isoforms, the log2(FC) was −1.03 and the expression was lower in tumors compared with control samples (p < 0.0001) (**Figure 1**). All data are listed in **Supplementary Table 1**.

**Figure 2:**
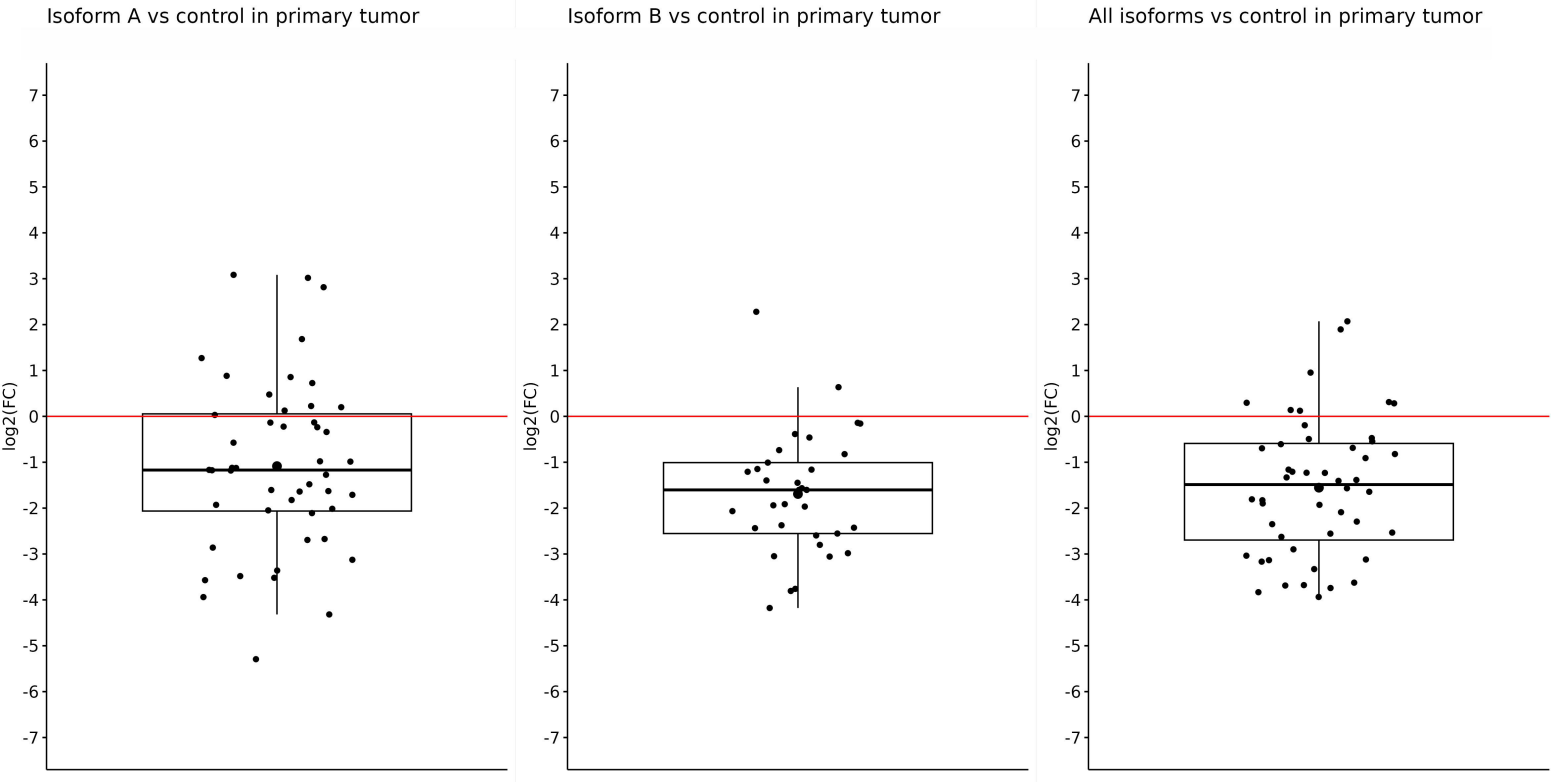
Expression of *OCT4A*, *OCT4B*, and all *OCT4* isoforms in primary tumors compared with control samples. In the artwork, we can see the significantly downregulated expression of all tested isoforms in primary tumors compared with control samples. The log2(FC) values are −1.08 for OCT4A, −1.69 for OCT4B, and −1.55 for all OCT isoforms.

After getting these results, we tried to determine whether the primary or metastatic samples were responsible for such reduced expression of individual isoforms in tumor samples or if it was a result of a mutual effect. Thus, subsequently, we determined the changes in the expression in primary and metastatic tumors compared with the control separately. In primary tumors, we also observed significantly reduced expression of all tested isoforms. For the *OCT4A* isoform, the log2(FC) value was −1.08, referring to reduced expression in primary tumors compared with control samples (*p* = 0.0002). The expression of *OCT4B* isoforms was the lowest in primary tumors, and compared with the control samples, the log2(FC) value was −1.69 (*p* < 0.0001). Furthermore, we obtained statistically significant results also for the remaining isoforms. For all *OCT4* isoforms, the log2(FC) value was −1.55, which means decreased expression in primary tumors compared with controls (*p* < 0.0001) (**Figure 2**).

**Figure 3:**
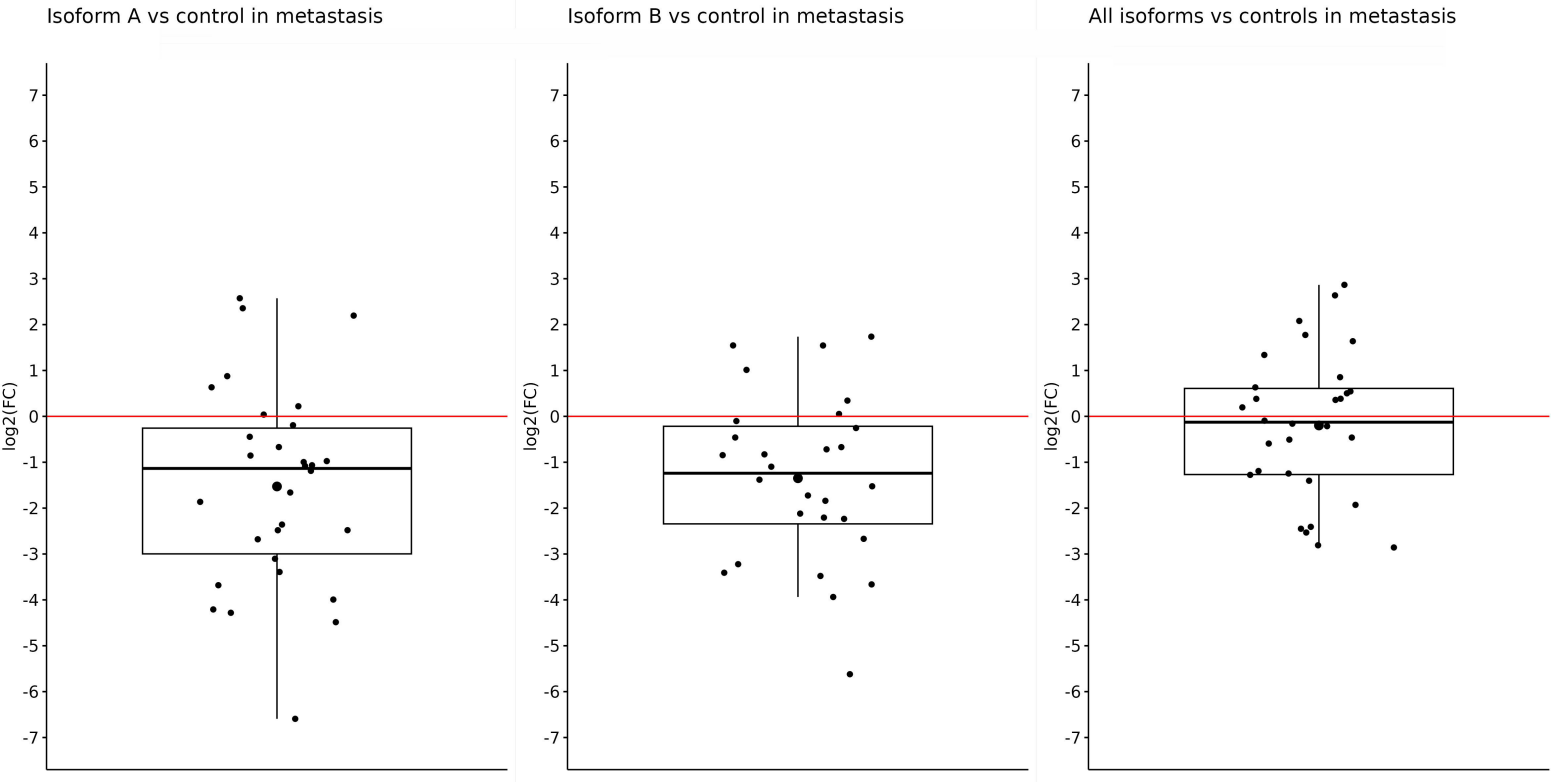
Expression of *OCT4A*, *OCT4B*, and all *OCT4* isoforms in metastases compared with control samples. When comparing metastases with controls, a significantly reduced expression was observed for only OCT4A (log2(FC) = −1.53) and OCT4B isoforms (log2(FC) = −1.35). For all OCT4 isoforms, the log2(FC) value was the lowest (−0.2) and without statistical significance.

Similar results were also obtained in metastatic samples; in other words, the expression of *OCT4A* and *OCT4B* isoforms was significantly downregulated in tumor samples compared with controls. Both the *OCT4A* and *OCT4B* isoforms had statistically significant results. The expression of OCT4A was reduced (log2(FC) = −1.53) in liver metastasis compared with control tissues (*p* = 0.0006). For OCT4B, the log2(FC) value was −1.35, indicating a lower expression in metastasis (*p* = 0.00051). On the other hand, although the log2(FC) for all *OCT4* isoforms was −0.2 and indicated reduced expression in metastases compared with controls, this difference was not enough to be statistically significant (*p* = 0.5) (**Figure 3**).

**Figure 4:**
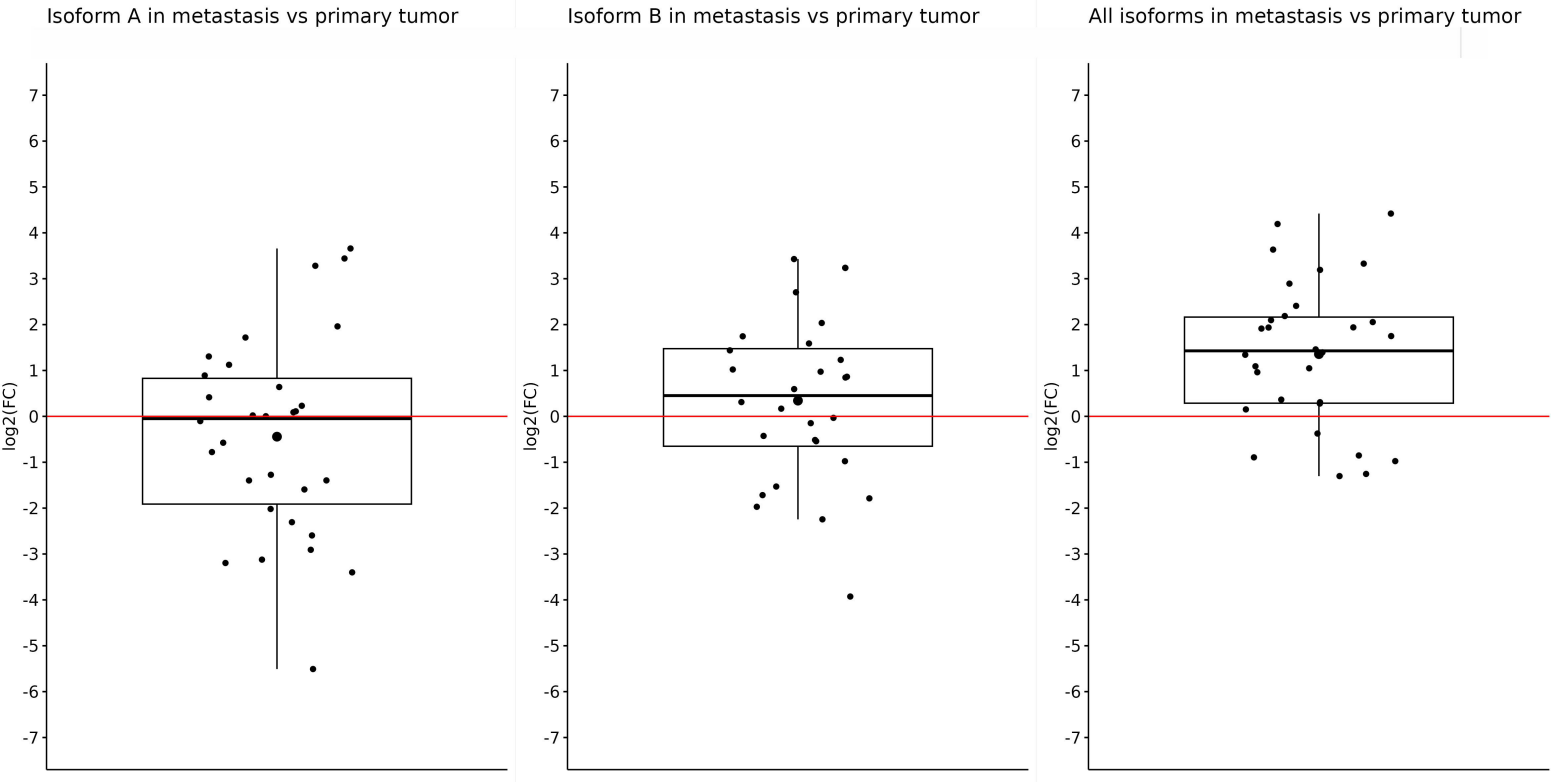
Expression of *OCT4A*, *OCT4B*, and all *OCT4* isoforms in metastases compared with primary tumor samples. Comparison of the expression in metastases and primary tumors proved only one statistically significant result, which showed us that the expression of all OCT4 isoforms is significantly upregulated in metastasis (log2(FC) = 1.357). For the OCT4A and OCT4B isoforms, there were slight downregulation and upregulation (log2(FC) = −0.44 and 0.34, respectively).

After that, we did an expression comparison in primary and metastatic tumors. Surprisingly, the expression of *OCT4A* and *OCT4B* was not significantly different in metastases and primary tumors, and log2(FC) −0.44 and 0.34 did not show statistically significant results (*p* = 0.27 and *p* = 0.33, respectively). Nonetheless, the expression of *OCT4A* was lower in metastases compared with primary tumors. On the contrary, *OCT4B* isoforms were overexpressed in liver metastases. We obtained the only statistically significant result in the case of all *OCT4* isoforms which were significantly overexpressed in metastases compared with primary tumors (log2(FC) = 1.357; *p* < 0.0001) (**Figure 4**).

So, in the case of *OCT4A*, the most downregulated expression was observed in metastatic tumors. Regarding the *OCT4B* isoforms, the most downregulated expression was also detected in metastases, too. In the case of all *OCT4* isoforms, when comparing metastases and primary tumors, the expression was significantly upregulated in metastatic samples. Data are available on data-mendeley.com/datasets (42).

### 3.2 Regression analysis

We further evaluated the relationship between the isoform expression and several clinical parameters, for instance, cancer type, tumor side, clinical, and TNM stage. Comparison of the tumor samples (primary + metastatic) with the control showed a correlation between significantly downregulated expression of all OCT4 isoforms and primary tumors (*p* = 0.001). Regression analysis also revealed the relationship between significantly reduced expression of all *OCT4* isoforms and left-sided tumors (*p* = 0.030). Other correlations regarding the expression of certain isoforms and the type or side of tumors were not observed.

### 3.3 TCGA results

We have downloaded the STAR-processed RNA sequencing level 1 data of 671 samples (colorectal tumors: *n* = 615, metastases: *n* = 11, unpaired samples from normal tissue: *n* = 45) from the Cancer Genome Atlas Consortium Portal and used them to extract transcripts per kilobase million (TPM) counts for *POU5F1* (Ensemble ID ENSG00000204531.20). POU class 5 homeobox 1 has seven splice variants, which unfortunately are not discriminated by level 1 data. The level of gene expression in normal, tumor, and metastatic tissues is summarized in **Supplementary Table 3** and **Supplementary Figure 2**. The TCGA results (**Supplementary Table 3**) indicate a rise in the expression of the *POU5F1* gene from non-tumor samples to primary tumors and subsequently to metastases, although without statistical significance. Given the low number of observations for two out of three groups and the skewness toward higher TPM values, it is inconclusive at any significance level to say that there is an increased expression from normal to primary tumors and to metastases, although there might be some trend in this direction. Welch’s *t*-test was used for pairwise testing of the null hypothesis that the population means of TPM in the normal, tumor, and metastatic groups are the same. Contrary to our previous expectations, in no tested combination did we reach the statistical significance at the level of <0.05 (normal *vs*. tumor: *p* = 0.17; normal *vs*. metastatic: *p* = 0.05, tumor *vs*. metastatic tissue: *p* = 0.06), probably owing to the high number of outliers.

## 4 Discussion

It is well known that the *OCT4A* isoform is expressed in numerous cell types including ES, EC, and cancer cell lines. Based on very little evidence about its expression in adult tissues (12, 43) and the study of Feldman et al., who found out that *OCT4A* transcription is turned off during gastrulation due to its promoter and enhancer methylation (44), it was generally thought that *OCT4A* is not expressed in adult somatic tissues. The OCT4A was shown to be an irreplaceable factor for stem-like cell phenotype, maintaining pluripotency and self-renewal of the stem cells and also for precise embryonic development, making it one of the most popular transcription factors ever (45–48). At present, a large number of articles demonstrate the presence of *OCT4A* in cancer stem cells (CSCs), and it is thought that *OCT4A* is responsible for stem-like cell properties of the cancer cells such as self-renewal, resistance, and the possibility to give rise to progenitor cells as well as epithelial–mesenchymal transition (19, 49). These assumptions make it a suitable target for treatment (50). In the past, it was proven that downregulation of *OCT4* expression resulted in inhibited tumorigenesis, reduced drug resistance, and induced G2/M phase arrest (51). Furthermore, *OCT4* appears to play a role in the angiogenesis and conversion of human fibroblasts to functional endothelial cells (52–54), and its expression was confirmed in all 13 CRC cell lines established from patients with both primary and metastatic tumors (55). Altogether, it appears that *OCT4A* contributes to tumor initiation, cancer growth, metastasis, and therapy resistance (56).

To date, 10 different *OCT4* isoforms have been identified at the RNA level, but not all are translated into protein (15). At the protein level, we can distinguish *OCT4A* and *OCT4B* isoforms which differ in their exon composition, nucleotide sequence, cell localization, and properties (11) (**Supplementary Table 2**). Due to the inability to bind to DNA, the OCT4B isoform is unable to regulate gene expression, and therefore, the role of OCT4B isoforms in cells remains unknown and unclear (18). Even though there are numerous *OCT4B* transcripts, the existence of only three protein isoforms has been confirmed (57). OCT4B proteins have been shown to play a role in stress response in two different ways. OCT4B-190 protects cells against apoptosis after heat shock (17), and in contrast, OCT4B-265 promotes apoptosis in reaction to genotoxic stress through the p53 signaling pathway (58).

In our study, we demonstrated an expression pattern of different *OCT4* isoforms in different sample types, as well as the relationship between isoform expression and several clinical parameters.

Comparing the expression of all determined isoforms (A, B, and all isoforms) in the control and tumor samples, regardless of the type, we observed significant overexpression in the control samples (**Figures 1**–**3**). Distinct results were presented by Liu et al. (59) who found out that *OCT4* was overexpressed in tumor tissue compared with their matched normal counterparts of CRC. However, the authors did not distinguish primary and metastatic samples nor the expression of individual isoforms because they used primers specific for *OCT4A* as well as *OCT4B* isoforms (59). On the other hand, similar results for the expression of all *OCT4* isoforms, such as reduced gene expression in tumor tissue compared with the control and regardless of the isoforms, were also obtained in breast cancer (60).Aside from the other isoforms, *OCT4A* also had a higher expression in the control samples. On the other hand, the most reduced expression was detected in metastases compared with control samples, and the lowest difference was observed in the comparison of primary tumors with control tissues. In earlier investigations, devoted to *OCT4A* expression, the authors pointed out the possible distortion of the results due to the existence of numerous *OCT4* pseudogenes, so we should also consider the fact that our expression data may be influenced by pseudogenes expression (61–63). To date, eight *OCT4* pseudogenes have been identified and the transcription of these pseudogenes can have a confusing effect on research and knowledge of the *OCT4* gene expression (64). On the contrary, Saha et al. published results in which the expression of several *OCT4* pseudogenes has the same trend compared with the *OCT4* expression which indicates that pseudogene expression should not have an impact on the overall direction of expression (60). It could be precisely pseudogenes that are responsible for such high *OCT4A* expression, but even though our results indicate that *OCT4A* expression is the highest in the control samples, our raw expression data demonstrated that *OCT4A* expression is not as high as it can be seen in the controls (average Ct approximately 31). We also observed that the expression of *OCT4A* was mildly decreased in metastases compared with primary tumors. We suppose that it is related to the change in *OCT4B* expression. Li et al. (65) found out that *OCT4B* functions as a non-coding RNA, modulating *OCT4A* expression by competitive binding with microRNAs. This may highlight the role of *OCT4B* in miRNA regulation of *OCT4A* expression (65). Hypothetically, this could be the reason why the expression of *OCT4A* was reduced in primary tumors, and with the mild increase in *OCT4B* expression, we observed a little but statistically non-significant decrease in *OCT4A* expression in metastases.

We also observed significantly upregulated expression of all *OCT4* isoforms in metastasis compared with primary tumors. Such increased expression may be caused by using a probe that specifically recognizes a mutual exon shared by all of the already identified *OCT4* isoforms. Thus, we were probably able to detect the expression of not only the *OCT4A* and *OCT4B* isoforms but also fewer known isoforms such as *OCT4C* and *OCT4D*, whose existence at the protein level has not yet been confirmed (15, 16). Several research groups have published results that emphasize the role of *OCT4* in the aggressive behavior of CRC and its contribution to forming liver metastasis in CRC, especially *OCT4* in the high-expression group, which is consistent with our results (66–68). Furthermore, *OCT4* expression was denoted as an independent prognostic biomarker for predicting worse disease-specific survival and overall survival in CRC (69). On the other hand, the expression of all *OCT4* isoforms in both primary and metastatic samples than in control samples was significantly downregulated. Similar results, such as reduced gene expression of *OCT4* in tumor tissue compared with control and regardless of the isoforms, were also obtained from breast cancer (60).

For the *OCT4B* isoforms, there is a typical expression in various non-pluripotent cell types and differentiated tissues at different levels based on a specific isoform. There is also unequivocal evidence that *OCT4A* is also expressed in adult human stem cells and differentiated somatic cells, in addition to pluripotent cells, but at a much lower level (14). Surprisingly, *OCT4B1* has a similar expression pattern to *OCT4A* (18). As a result of determining the expression of all *OCT4B* isoforms together, we were not able to designate which isoform had a higher expression and vice versa. In primary tumors, the expression of the *OCT4B* isoforms was significantly reduced compared with controls. When compared with metastases, the expression was slightly reduced but without a statistically significant result.

Although we used data from the TCGA and despite our efforts, we were not able to confirm in the TCGA dataset our findings of reduced *POU5F1* gene expression in tumor samples compared with non-tumor controls at a statistically significant level. The TCGA results (**Supplementary Table 3**) indicate a non-significant trend of the increase of the *POU5F1* expression from normal tissue to primary tumor and to metastases. Our analyses demonstrate only a significant increase in gene expression solely between the primary and metastatic samples. In contrast, we have observed a decrease in gene expression in tumor samples relative to non-tumor samples. Although it would have been ideal to have paired TCGA data, the number of non-cancerous samples available for comparison was rather limited. To avoid any contradictions in our research, we believe that it is important to conduct further validation studies with a larger sample size and better access to the TCGA data. It will also be necessary to differentiate between individual OCT4 isoforms, which were not distinguished in many previous studies, to obtain more conclusive results on the gene expression of *POU5F1* and its isoforms. These efforts will provide a better understanding of the role of *POU5F1* in colorectal cancer.

The role of *OCT4* expression as a prognostic marker, as well as its role in metastatic CRC, has already been explored. Patients with high *OCT4* expression had a poorer prognosis, making it a potential marker for the diagnosis and assessment of the prognosis of CRC. In addition, the results also indicate that *OCT4* expression was correlated with clinical stage, tumor grade, metastasis, and TNM stage (69–71). In our study, we did not observe any correlation between *OCT4* isoform expression and clinical or TNM tumor stage. The authors of the studies mentioned above did not recognize between individual *OCT4* isoforms which may cause inconsistency in the results. Our results also suggest a correlation between reduced expression of all *OCT4* isoforms and both primary and left-sided tumors. On the other hand, Talebi et al. did not observe *OCT4* expression in any of the tissues tested (normal, polyp, and cancer tissue) and concluded that the diagnostic power of the *OCT4* gene is not enough to identify cancer (72). Even though the exact role of *OCT4B* isoforms in the cell is still under investigation, several studies indicate that in some way they may contribute to the properties of cancer cells, such as invasion, having antiapoptotic properties, and resistance to chemotherapy (73, 74). In addition, Gazouli et al. confirmed the expression of the *OCT4B* and *OCT4B1* isoforms in CRC samples and observed that the level of *OCT4B1* mRNA was correlated with poorly and moderately differentiated CRC and with the progression of cancer stage (62). In our study, no correlation was detected between *OCT4B* expression and clinical data as well as the type or side of the tumor or TNM stage. Another research group showed that *OCT4B1* has a potential role in regulating the self-renewal of colorectal CSC through its involvement in epithelial–mesenchymal transition (75). Furthermore, Simó-Riudalbas et al. demonstrated a pro-oncogenic effect of *OCT4B1* through its association with protein kinases and subsequent activation of intracellular signaling events as well as cytoskeletal rearrangements (76). All these findings can signify a potential role of *OCT4B*, especially *OCT4B1* as a marker of tumor-initiating cells or CSCs, and not only *OCT4A* but all *OCT4* isoforms might play a significant role in carcinogenesis.

Based on previous results as well as our results, the expression level of *OCT4* isoforms could be a useful tool not only for diagnosis, especially metastatic disease, but also for prognosis prediction. As the results of the TCGA analysis and our own analyses are not fully consistent, further studies will be needed to clarify these differences. Thus, these results emphasize the importance of precise characterization of individual *OCT4* isoforms whether transcriptional or protein, as well as their expression patterns in colorectal cancer.

## 5 Conclusion

Unlike previous reports, we found out that the expression of *OCT4A*, *OCT4Bs*, and all *OCT4* isoforms is significantly reduced in primary tumors and metastases compared with control samples. On the other hand, the expression of all *OCT4* isoforms was significantly upregulated in metastases compared with primary tumors, which was not caused by the upregulation of the *OCT4A* isoform and only partially by the *OCT4B* isoform, emphasizing the role of less-known isoforms in metastatic CRC. Furthermore, the reduced expression of all *OCT4* isoforms was correlated with primary and left-sided tumors. Based on these results, we supposed that the expression rate of all *OCT4* isoforms can be related to the cancer type and side, as well as to liver metastasis. However, further studies are required to investigate the detailed expression patterns and significance of individual *OCT4* isoforms in carcinogenesis.

## Data availability statement

The datasets presented in this study are available on https://data.mendeley.com/datasets/2c5296ytbr (42).

## Ethics statement

The studies involving human participants were reviewed and approved by the Ethics Committee of the Jessenius Faculty of Medicine in Martin, Comenius University in Bratislava by decision number 1863/2016. The patients/participants provided their written informed consent to participate in this study.

## Author contributions

ET was responsible for the experimental design, performed the experiments, and wrote the manuscript. ZL secured the financial funding, was responsible for the experimental design, supervised the data analysis and interpretation, and critically reviewed and revised the manuscript drafts. VH and ZL assisted in conducting the experiments. MG performed the bioinformatic analyses and generated the figures. MH and MKo performed the gene analysis from the TCGA raw reads and wrote the TCGA part of the manuscript. MKa and JMa performed the histopathological evaluation. EK and PM collected the patients’ samples. JMi and LL critically reviewed and revised the manuscript. All authors contributed to the article and approved the submitted version.

## Supplementary material

Supplementary Figure 1 Areas within the *POU5F1* gene which are recognized by specific TaqMan probe.

Supplementary Figure 2 Gene expression of *POU5F1* based on Transcript Per Kilobase Million (TPM) counts from RNA sequencing experiments. Conformity to the Gaussian distribution was assessed and was subsequently rejected in each group, owing to the substantial enrichment of samples with high expression (Shapiro-Wilk test: normal tissue: P = 2.901e-10, tumor tissue: P < 2.2e-16, metastases: P = 0.0009742).
