# Supplementary figures and images for: Expression of OCT4 isoforms is reduced in primary colorectal cancer
Source: Front Oncol. 2023 Jun 20;13:1166835. doi: 10.3389/fonc.2023.1166835 (PMC10319064; doi:10.3389/fonc.2023.1166835)

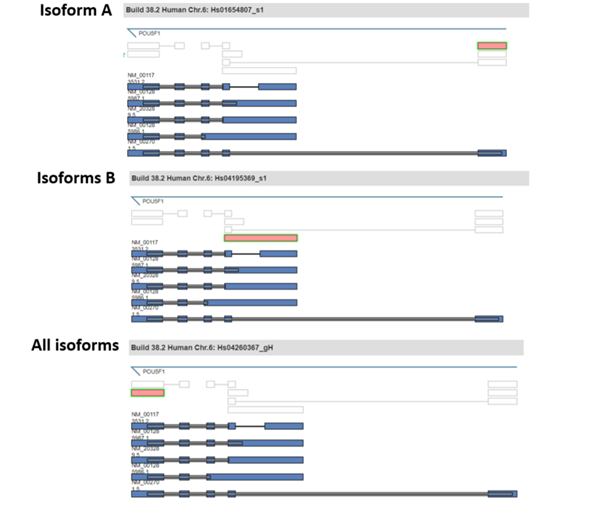

Supplement: Supplementary Figure 1 — Areas within the POU5F1 gene which are recognized by specific TaqMan probe. [file Image_1.png]

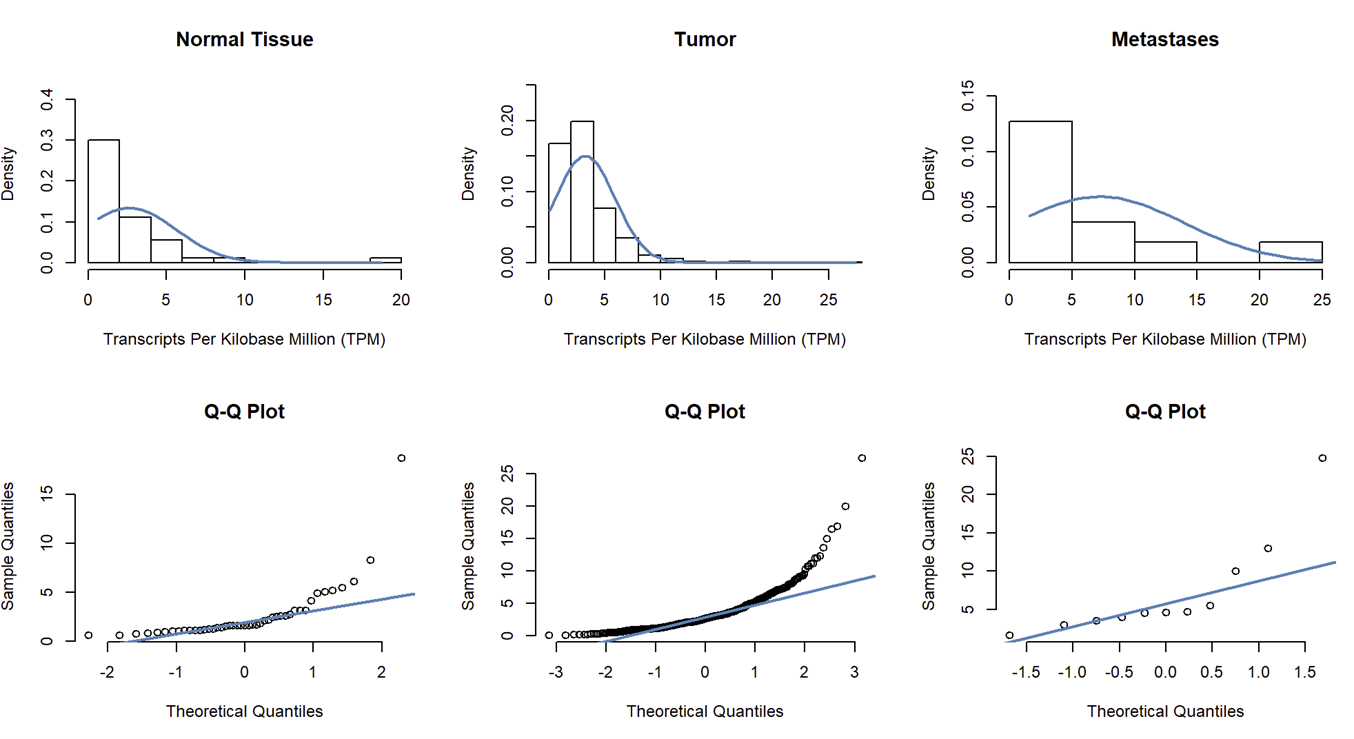

Supplement: Supplementary Figure 2 — Gene expression of POU5F1 based on Transcript Per Kilobase Million (TPM) counts from RNA sequencing experiments. Conformity to the Gaussian distribution was assessed and was subsequently rejected in each group, owing to the substantial enrichment of samples with high expression (Shapiro-Wilk test: normal tissue: P = 2.901e-10, tumor tissue: P < 2.2e-16, metastases: P = 0.0009742). [file Image_2.png]
