# Supplementary material for: Expression of OCT4 isoforms is reduced in primary colorectal cancer
Source: Front Oncol. 2023 Jun 20;13:1166835. doi: 10.3389/fonc.2023.1166835 (PMC10319064; doi:10.3389/fonc.2023.1166835)
Supplement: Supplementary file 3 [file Table_1.docx]

Supplementary table 1: Overview of results from statistical analysis.

|  | All primary and metastatic tumor samples compared to adjacent non-tumor tissue | | | | | |
| --- | --- | --- | --- | --- | --- | --- |
|  | Log2(FC) | Expression | | P value | | Regression analysis |
| **OCT4A** | -1.25 | Downregulated | | P<0.0001 | | No correlation |
| **OCT4B** | -1.53 | Downregulated | | P<0.0001 | | No correlation |
| **All OCT4 isoforms** | -1.03 | Downregulated | | P<0.0001 | | Type (primary) p= 0.001  Side (left) p= 0.03 |
|  | Primary tumor samples compared to adjacent non-tumor tissue | | | | | |
|  | Log2(FC) | Expression | | P value | | Regression analysis |
| **OCT4A** | -1.08 | Downregulated | | P=0.0002 | | Clinical stage II (P=0.026) |
| **OCT4B** | -1.69 | Downregulated | | P<0.0001 | | No correlation |
| **All OCT4 isoforms** | -1.55 | Downregulated | | P<0.0001 | | No correlation |
|  | Metastatic tumor samples compared to adjacent non-tumor tissue | | | | | |
|  | Log2(FC) | | Expression | P value | Regression analysis | |
| **OCT4A** | -1.53 | | Downregulated | P=0.0006 | No correlation | |
| **OCT4B** | -1.35 | | Downregulated | P=0.00051 | No correlation | |
| **All OCT4 isoforms** | -0.2 | | Downregulated | P=0.5 | No correlation | |
|  | Metastatic tumor samples compared to primary tumor samples | | | | | |
|  | Log2(FC) | | Expression | P value | Regression analysis | |
| **OCT4A** | -0.44 | | Downregulated | P=0.27 | No correlation | |
| **OCT4B** | 0.34 | | Upregulated | P=0.32 | No correlation | |
| **All OCT4 isoforms** | 1.357 | | Upregulated | P<0.0001 | No correlation | |
