# Supplementary material for: Expression of OCT4 isoforms is reduced in primary colorectal cancer
Source: Front Oncol. 2023 Jun 20;13:1166835. doi: 10.3389/fonc.2023.1166835 (PMC10319064; doi:10.3389/fonc.2023.1166835)
Supplement: Supplementary file 4 [file Table_2.docx]

Supplementary table 2: Overview of individual *OCT4* isoforms.

| **Isoform** | **Transcript** | **Protein** | **Cellular localization** | **Function** |
| --- | --- | --- | --- | --- |
| OCT4A | NM_002701 (11) | 360 AA (11). | Exclusively in nucleus (59). | Transcription factor (responsible for stemness properties such as self-renewal and pluripotency maintenance of embryonic stem and embryonic cancer cells) (60). |
| OCT4B | NM_203289 (11) | 265, 190 and 164 AA (alternative translation initiation) (39). | Diffusely localized in both cytoplasm and nucleus (17) | Stress response (protection of cells against apoptosis after heat shock; promotion of apoptosis after genotoxic stress) (17, 40). |
| OCT4B1 | EU518650 (12) |  |  |  |
| OCT4B2 | KJ700311 (13a) | Predicted 164 AA (10). | Predicted localization in both cytoplasm and nucleus (17) |  |
| OCT4B3 | KJ624996 (13b) |  |  |  |
| OCT4B4 | KM188057 (13c) |  |  |  |
| OCT4B5 | KM188058 (15). |  |  |  |
| OCT4C  OCT4C1 | AB971680, AB971681 (16). |  |  |  |
| OCT4D | KY781167 (14). | Predicted 265 AA |  |  |

References:

(13b) Poursani EM, Mehravar M, Mohammad Soltani B, Mowla SJ, Trosko JE. A Novel Variant of OCT4 Entitled OCT4B3 is Expressed in Human Bladder Cancer and Astrocytoma Cell Lines. Avicenna J Med Biotechnol (2017) 9:142-145. DOI is not available.

(13c) Poursani EM, Mehravar M, Soltani BM, Mowla SJ. Novel variant of OCT4B4 is differentially expressed in human embryonic stem and embryonic carcinoma cells. Gene (2017) 627:369-372. DOI: 10.1016/j.gene.2017.06.032.

(59) Pan G, Qin B, Liu N, Schöler HR, Pei D. Identification of a nuclear localization signal in OCT4 and generation of a dominant negative mutant by its ablation. J Biol Chem (2004) 35:37013–20. DOI: 10.1074/jbc.M405117200.

(60) Wang X, Dai J. Concise Review: Isoforms of OCT4 Contribute to the Confusing Diversity in Stem Cell Biology. Stem Cells (2010) 5:885–93. DOI: 10.1002/stem.419.
