# Supplementary material for: Expression of OCT4 isoforms is reduced in primary colorectal cancer
Source: Front Oncol. 2023 Jun 20;13:1166835. doi: 10.3389/fonc.2023.1166835 (PMC10319064; doi:10.3389/fonc.2023.1166835)

Supplementary table 3: Gene expression of POU5F1 based on Transcript Per Kilobase Million (TPM) counts from RNA sequencing experiments.

| **Sample Type** | **0%**  **Minimum** | **25%** | **50%**  **Median** | **Mean** | **75%** | **100%**  **Maximum** | **SD** |
| --- | --- | --- | --- | --- | --- | --- | --- |
| Normal | 0.6314 | 1.1400 | 1.6300 | 2.6312 | 2.7300 | 18.7232 | 2.9651 |
| Tumor | 0.1039 | 1.5650 | 2.6500 | 3.2622 | 4.1035 | 27.4349 | 2.6403 |
| Metastatic | 1.6370 | 3.7260 | 4.6330 | 7.1996 | 7.7550 | 24.8424 | 6.7000 |


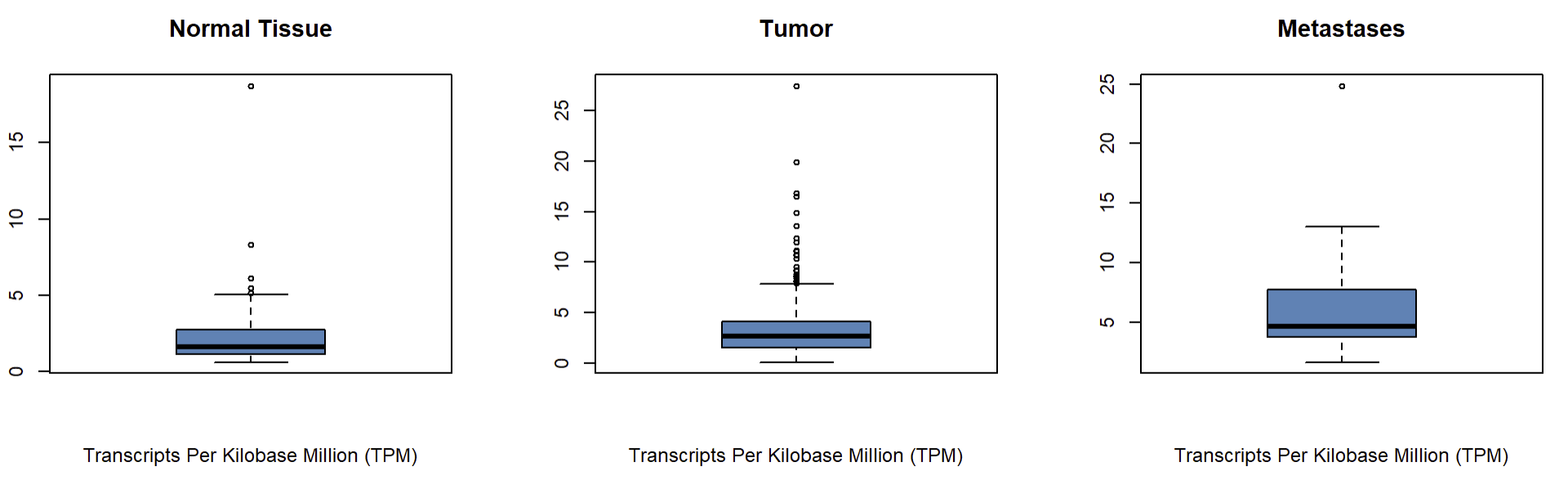

Supplement: Supplementary file 5 [file Table_3.docx]
